# BR-YOLOv9: a multi-scale fusion framework for robust weed recognition in rice paddy fields

**DOI:** 10.3389/fpls.2026.1842189

**Published:** 2026-07-08

**Authors:** Zhiwei Wang, Lin Zhou, Zihan Yue, Lantian Ren, Bo Yao

**Affiliations:** Anhui Science and Technology University/Anhui Engineering Research Center for Smart Crop Planting and Processing Technology, Chuzhou, China

**Keywords:** BR-YOLOv9, intelligent weeding, multi-scale feature fusion, paddy-field weed detection, RepFEL

## Abstract

To address the low accuracy and limited generalization of intelligent weed recognition under complex paddy-field conditions involving illumination variation, vegetation occlusion, multi-scale targets, and complex background interference, this study proposes an improved YOLOv9-based weed detection model, termed BR-YOLOv9. First, a weighted bidirectional feature pyramid network (BiFPN) was introduced into the neck network of YOLOv9 to strengthen cross-scale feature fusion and improve the representation of small and multi-scale weed targets. Subsequently, the original RepNCSPELAN4 module was reconstructed, and a novel RepFEL module was designed by integrating feature enhancement and a large selective kernel mechanism to improve fine-grained feature extraction and suppress background interference. Experimental results showed that BR-YOLOv9 achieved mAP@0.5 and mAP@0.5:0.95 values of 98.21% and 95.97%, respectively, representing improvements of 2.03 and 3.71 percentage points over the original YOLOv9 model. In the generalization experiments, BR-YOLOv9 was further evaluated under multiple representative field scenarios, including close-up, long-distance, high-illumination, low-illumination, and multi-target conditions. The results showed that BR-YOLOv9 maintained stable detection performance across different imaging distances, illumination conditions, and target densities, demonstrating stronger robustness and generalization capability in complex agricultural environments. The proposed model provides a robust visual perception method for paddy-field weed detection and offers technical support for precision weed management and intelligent weeding equipment.

## 1 Introduction

Rice is a major staple crop worldwide (Duan et al., 2024). In paddy fields, weeds vie with rice for essential resources, including light, nutrients, and space, and may also serve as reservoirs for pests and diseases, thereby adversely affecting both yield and quality (Xu et al., 2024). At present, commonly used weed control methods in agricultural production, including manual weeding, mechanical weeding, and chemical weeding, each have their own limitations. Manual weeding requires substantial labor input and is generally inefficient, mechanical weeding shows poor adaptability, and chemical weeding, although highly efficient, is associated with herbicide waste and environmental risks (Upadhyay et al., 2024).With the development of precision agriculture and variable-rate spraying technology, site-specific and quantitative herbicide application based on the spatial distribution, species, and density of field weeds has become a key approach for reducing herbicide use and improving weed control efficacy. Its effective implementation depends on the rapid and accurate recognition of rice and weeds under natural field conditions (Farooque et al., 2023).

Early field weed identification studies typically used handcrafted features, including color, texture, and morphology, in combination with conventional classifiers to separate crops from weeds (Espejo-Garcia et al., 2020). However, these methods show limited generalization under illumination variation, shadow interference, occlusion, and complex field backgrounds (Wang et al., 2019). With the rapid development of deep learning, convolutional neural networks (CNNs) have greatly improved feature representation capability and have been widely used in agricultural target recognition tasks. Existing deep-learning-based weed recognition methods can generally be divided into segmentation-based and detection-based approaches. Segmentation-based methods, such as FCN and DeepLab, can provide pixel-level weed region extraction, but they usually require dense annotations and relatively high computational cost, which may limit their efficiency in real-time field applications (Hu et al., 2024).

In contrast, object detection methods directly predict the category and location of weed targets using bounding boxes, making them more suitable for practical weed monitoring and intelligent weeding equipment. Representative detection algorithms include two-stage detectors, such as Faster R-CNN, and one-stage detectors, such as SSD and the YOLO series. Faster R-CNN introduces a region proposal network (RPN) to generate candidate regions and then performs classification and bounding-box regression, achieving high detection accuracy but relatively low inference efficiency (Liu et al., 2020). One-stage detectors eliminate the region proposal stage and directly perform dense prediction, thereby improving inference speed. Among them, the YOLO series has been widely applied in agricultural object detection because of its favorable balance between accuracy and real-time performance (Silva et al., 2024). In recent versions, YOLO-based detectors have continuously improved feature fusion, multi-scale detection, and lightweight deployment capability, making them suitable for complex field scenarios involving small targets, occlusion, illumination changes, and background interference. YOLOv9 further improves parameter utilization and gradient information transmission through the generalized efficient layer aggregation network (GELAN) and programmable gradient information (PGI), providing a strong baseline for paddy-field weed detection under complex conditions.

Representative studies on field weed identification have demonstrated that complex field environments, scale variation, and crop–weed similarity remain major challenges for robust recognition. Mu et al. integrated a feature pyramid network (FPN) into Faster R-CNN for weed seedling recognition under complex field backgrounds, indicating that multi-scale feature fusion is effective for detecting small weed targets and handling scale variation (Zhang et al., 2023). Yu et al. proposed an improved DeepLabv3+ model for soybean weed segmentation by using Swin Transformer as the backbone and introducing a convolutional block attention module (CBAM) after feature fusion, which improved pixel-level discrimination under dense and overlapping conditions (Yu et al., 2022). Peteinatos et al. systematically evaluated the applicability of CNNs for weed identification in multi-crop scenarios, including maize, sunflower, and potato, providing evidence for data-driven weed recognition across different crop types (Peteinatos et al., 2020). These studies demonstrate the effectiveness of deep learning in field weed recognition; however, segmentation-based methods generally require pixel-level annotations and high computational cost, while two-stage detectors may have limited inference efficiency for real-time field deployment.

In contrast, YOLO-based one-stage detectors provide a more practical solution for intelligent weeding equipment because they can simultaneously achieve object localization, classification, and real-time inference. Dang et al. proposed the YOLOWeeds benchmark and dataset and systematically compared multiple YOLO detectors, providing a reproducible framework for evaluating the trade-off between detection accuracy and speed in field multi-class weed detection (Dang et al., 2023). Compared with earlier YOLO versions, YOLOv9 further improves parameter utilization and gradient information transmission through the generalized efficient layer aggregation network (GELAN) and programmable gradient information (PGI), achieving a favorable balance between detection performance and computational efficiency (Wang et al., 2024). Therefore, YOLOv9 provides a suitable baseline for real-time paddy-field weed detection.

Nevertheless, directly applying YOLOv9 to paddy-field weed detection still faces challenges caused by multi-scale weed targets, illumination variation, different shooting distances, occlusion, and background texture noise (Shao et al., 2023). To address these limitations, BiFPN can be introduced to strengthen bidirectional cross-scale feature fusion and improve the representation of small and multi-scale weed targets (Tan et al., 2020). In addition, the large selective kernel (LSK) module can expand the adaptive receptive field and enhance the model’s ability to suppress background interference and capture discriminative weed features in complex paddy-field scenes (Shi et al., 2024; Li et al., 2025; Zhai et al., 2025). Therefore, integrating BiFPN and LSK into YOLOv9 is expected to improve the robustness and detection accuracy of weed recognition under natural paddy-field conditions.

In this study, the experimental dataset was constructed by combining a self-collected weed image dataset with a public dataset, and data augmentation was employed to enhance the model’s recognition performance. An improved YOLOv9 model was proposed. Specifically, BiFPN was incorporated into the backbone–neck network of YOLOv9 to perform weighted bidirectional multi-scale feature fusion, and a large selective kernel (LSK) module was integrated to improve fine-grained feature extraction and suppress background noise. Moreover, the original RepNCSPELAN4 module was reconstructed, and a novel RepFEL module was proposed to further improve weed detection accuracy under complex field conditions. The ablation studies and comparative experiments with multiple models showed that the improved BR-YOLOv9 model achieved more accurate weed identification in paddy fields and demonstrated stronger generalization capability than existing mainstream models. This study provides a more accurate and robust visual perception method for weed detection in paddy fields, offering technical support for the development of intelligent weeding and precision herbicide application systems.

## 2 Materials and methods

### 2.1 Image acquisition

**Figure 1:**
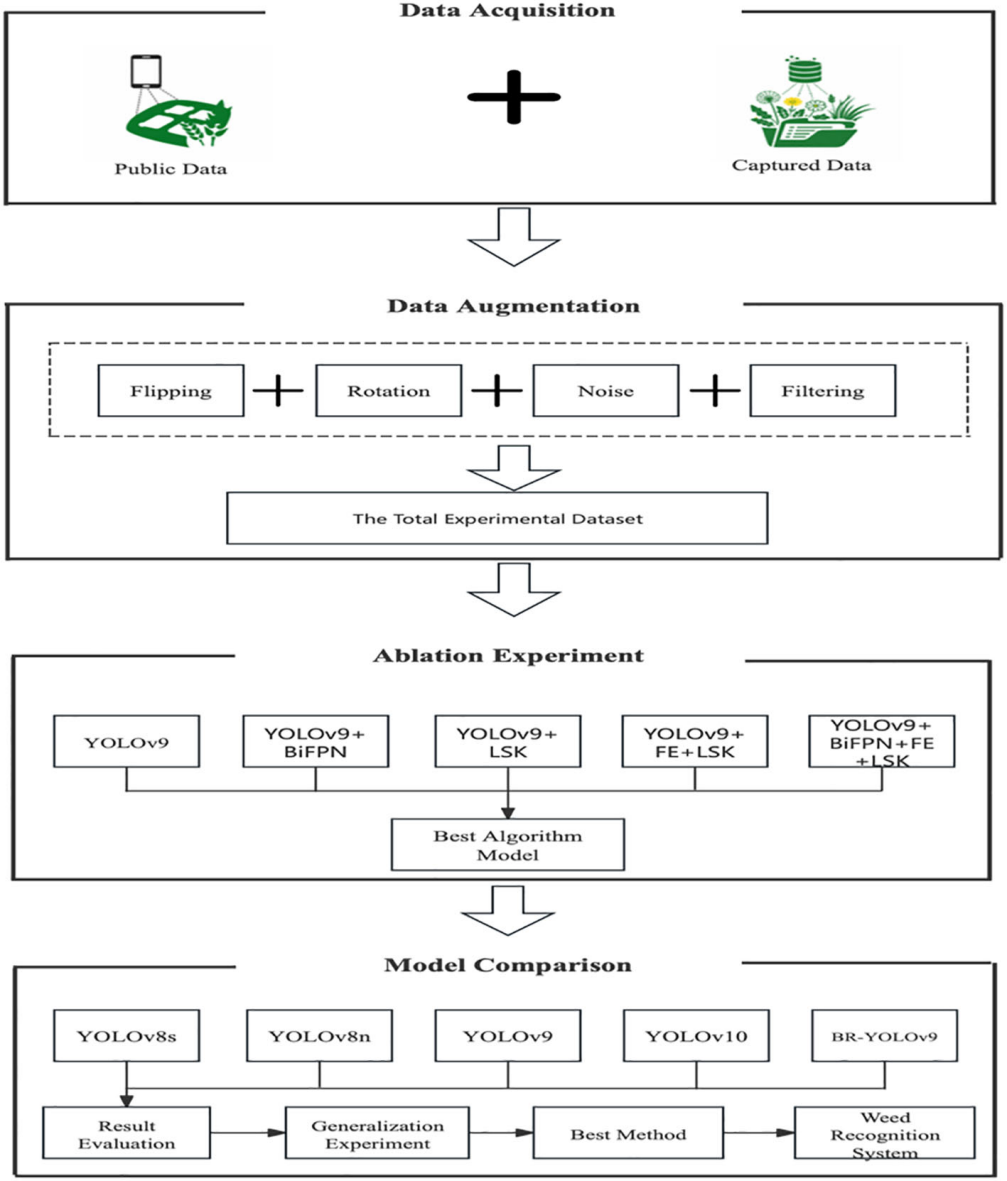
Overall technical workflow of this study.

**Figure 2:**
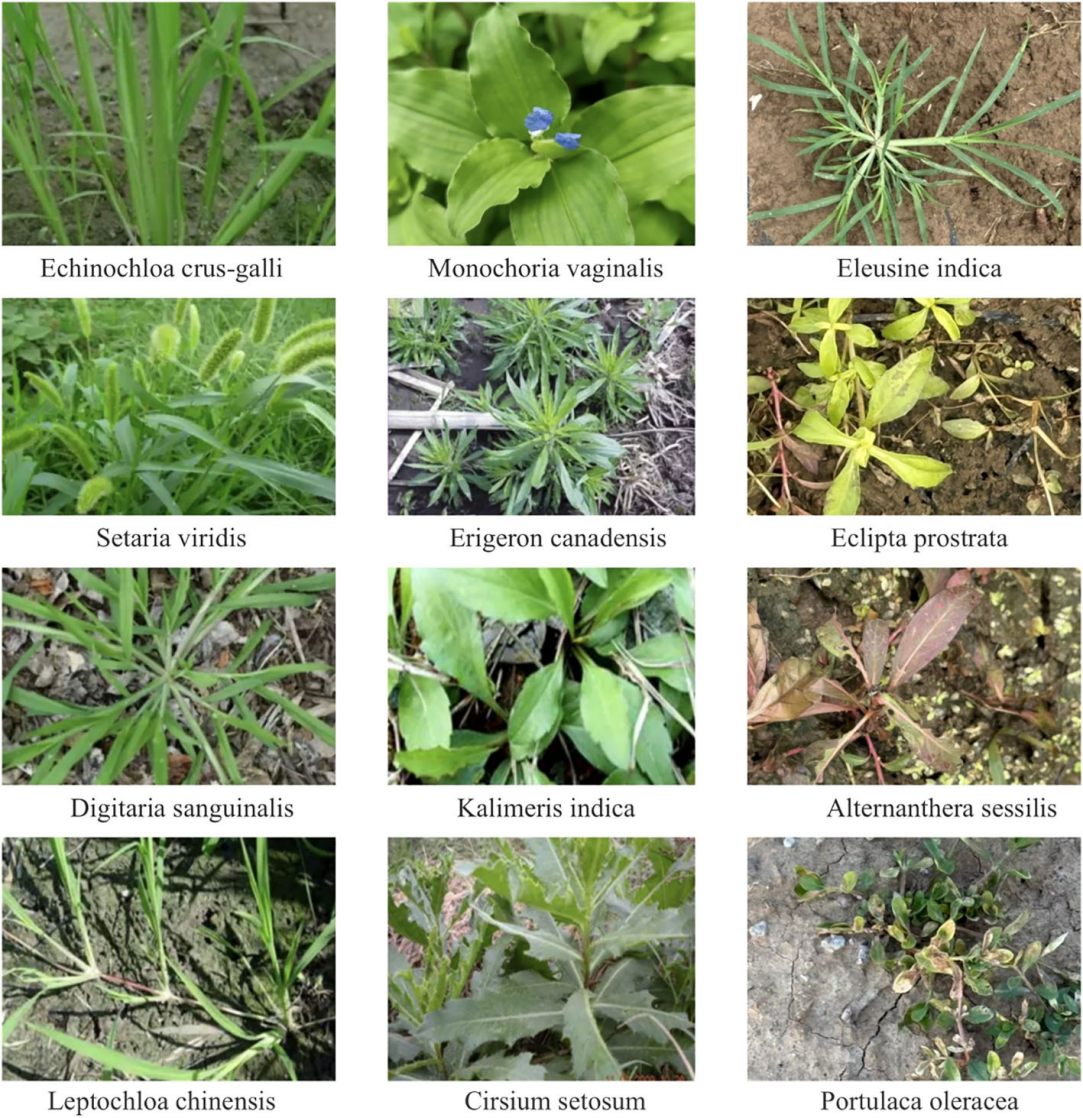
Weed images in rice paddy fields.

This study investigated weeds in paddy fields in Bengbu, Dingyuan County, and Fengyang County, Anhui Province, China. The overall technical workflow is shown in **Figure 1**. Image acquisition was completed on 2 August 2025. The collected weed categories included 12 typical paddy-field weeds, such as Eleusine indica, Eclipta prostrata, Alternanthera sessilis, and Portulaca oleracea, as shown in **Figure 2**. The dataset covered weeds occurring both before rice transplanting and during rice growth. The acquired images included complex field backgrounds under high-illumination and low-illumination conditions, as well as close-up, long-distance, and multi-target weed scenes. In total, 6,115 original images were collected in this study, of which 1,032 were collected from online sources and 5,083 were captured directly in the field. All images were acquired using a smartphone camera with a resolution of 48 megapixels. The image counts and label distribution for each weed category are summarized in **Table 1**.

**Table 1:** Weed species and sample distribution.

Name of weeds	Number of images	Number of labels
Eleusine indica	518	1534
Eclipta prostrata	502	1371
Alternanthera sessilis	507	1329
Portulaca oleracea	510	1549
Echinochloa crus-galli	502	1253
Setaria viridis	507	1344
Digitaria sanguinalis	512	1325
Leptochloa chinensis	515	1487
Monochoria vaginalis	506	1551
Erigeron canadensis	503	1453
Kalimeris indica	518	1311
Cirsium setosum	515	1253
Total	6115	16760

### 2.2 Dataset construction and experimental methodology

In this study, data augmentation strategies were applied to the training set to mitigate the adverse effects of complex background interference and improve the model’s generalization capability (Chen and Er, 2025). On the one hand, symmetric transformations, including rotation and flipping, were used to expand the diversity of target viewpoints and postures, thereby enriching scene representation in the samples. On the other hand, Gaussian noise and salt-and-pepper noise were incorporated to simulate weak image degradation commonly observed during field image acquisition. Specifically, Gaussian noise was used to approximate continuous low-intensity pixel disturbances caused by camera sensor noise, image compression, weak illumination, and slight imaging instability during handheld acquisition. Salt-and-pepper noise was used to approximate sparse local interference, such as small soil spots, water droplets, specular highlights on leaf surfaces, and tiny occlusions that may occur in paddy-field environments.

**Figure 3:**
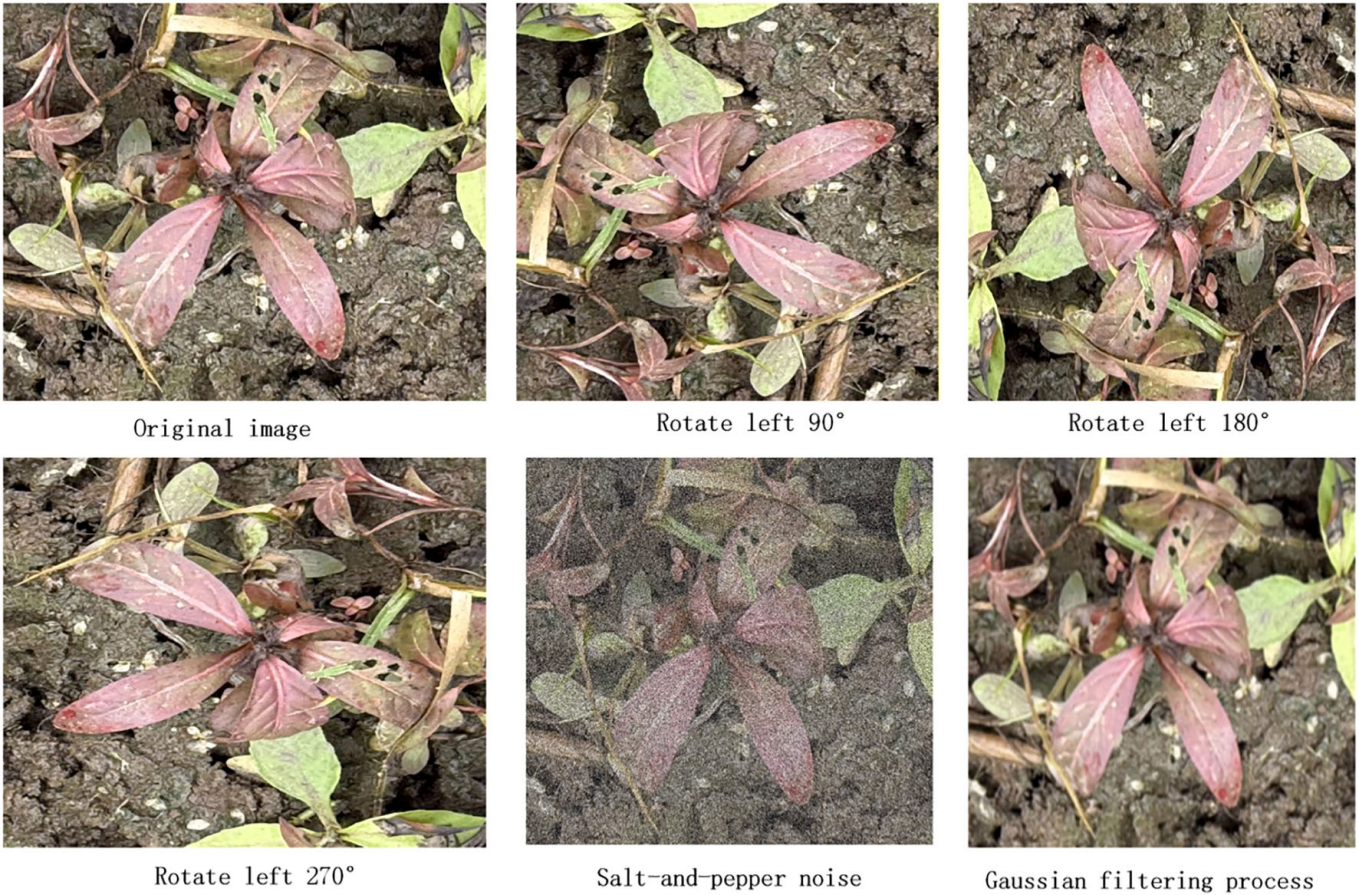
Visualization of data augmentation results.

To avoid introducing irrelevant or unrealistic noise, the noise intensity was strictly controlled within a mild range that did not affect the semantic interpretability of the targets. The augmented images were visually inspected to ensure that the morphological structure, target boundaries, and category labels of weed samples remained unchanged. Therefore, the noise augmentation was not intended to fully reproduce all complex physical disturbances in field environments, but rather to provide controlled pixel-level perturbations that improve the model’s tolerance to weak imaging noise and local image degradation. This strategy effectively avoided drift and distribution shift caused by excessive noise (Ramírez-Agudelo et al., 2025), thereby improving the model’s generalization capability and robustness while alleviating overfitting. The data augmentation procedure in this experiment was implemented in Python using PyCharm, with Python 3.11, OpenCV 4.12, and NumPy 2.2.6. **Figure 3** illustrates the augmentation results.

After the above augmentation procedures, the number of training samples increased from 6,115 to 18,345 images. The dataset partition followed two core principles. First, there was no overlap at the image level, ensuring the independence of each subset. Second, the class proportions were kept generally consistent to maintain a balanced data distribution. Ultimately, the dataset was partitioned into training, validation, and test sets at a ratio of 70%, 20%, and 10%, respectively. All augmented samples derived from the same original image were assigned exclusively to the training set, thereby preserving the independent and identically distributed characteristics of the validation and test sets and ensuring the objectivity and reliability of the evaluation results.

### 2.3 Experimental setup and evaluation criteria

To examine the detection performance of BR-YOLOv9 for weed recognition in paddy fields, model training was performed under a unified hardware and software environment. The hardware configuration consisted of an NVIDIA GeForce RTX 5070 Ti 16 GB GPU, an Intel Core i7-14650HX processor, 64 GB RAM, and 1 TB SSD storage. The software environment was based on the Windows 11 operating system. Python was used as the programming language, PyTorch 2.2.2 was adopted as the deep learning framework, and CUDA 12.8 was used for GPU acceleration. Weed images were annotated using LabelImg, and all images were recorded at a resolution of 480 × 640. Training was conducted for 300 epochs with the SGD optimizer at an initial learning rate of 0.01 to ensure parameter optimization and convergence.

A comprehensive evaluation of model performance was conducted using precision (P), recall (R), average precision (AP), mean average precision at an IoU threshold of 0.5 (mAP@0.5), and mean average precision over IoU thresholds from 0.5 to 0.95 with a step size of 0.05 (mAP@0.5:0.95).Precision indicates how accurately the model identifies weed samples, whereas recall represents its capacity to detect the actual weed samples present. Average precision reflected the discrimination model performance for different weed categories by calculating the area under the precision–recall curve for each class, mAP served as a comprehensive indicator for evaluating the overall model performance in the paddy-field weed detection task. The calculation process is as shown in Equations 1–5:

(1) $$\begin{array}{c} P=\frac{TP}{TP+FP} \end{array}$$

(2) $$\begin{array}{c} R=\frac{TP}{TP+FN} \end{array}$$

(3) $$AP=\int_{1}^{0}P(R)dR$$

(4) $$\begin{array}{c} mAP_{0.5}=\frac{\sum_{j=1}^{k}AP_{i}}{k(classes)}(IOU=0.5) \end{array}$$

(5) $$\begin{array}{c} mAP@0.5:0.95=\frac{\sum_{i=1}^{k}\sum_{t=0}^{9}AP_{i}}{10k(classes)}\;(IoU=0.5+0.0.5t) \end{array}$$

### 2.4 YOLOv9 architecture

YOLOv9 combines high detection accuracy with rapid response capability and has been widely applied in agricultural scenarios for the recognition and detection of plant diseases, insect pests, and weeds (Abulizi et al., 2024; Huang et al., 2024; Nguyen et al., 2025). This detection model achieves simultaneous improvements in accuracy and computational efficiency through two key advanced techniques, namely PGI and GELAN. While YOLOv9 demonstrates a favorable trade-off between accuracy and speed in object detection tasks, YOLOv9 may still face performance limitations in complex paddy-field environments, particularly under conditions involving multi-scale weeds, illumination variation, and changes in shooting distance. Therefore, further optimization of the model structure and feature representation is needed in combination with the specific characteristics of agricultural scenarios (Peng et al., 2022).

### 2.5 Weighted bidirectional feature pyramid network

In object detection research, achieving a balance between accuracy and efficiency has long been a central objective (Zhao et al., 2024). To achieve this goal, YOLOv9 adopts an integrated scheme combining FPN and PANet. Owing to its multi-level feature extraction capability, FPN supports the recognition of targets of different sizes and supplies the model with rich multi-scale information. PANet, in contrast, focuses on facilitating information flow across feature layers and further improving feature quality by strengthening interlayer interaction to compensate for the loss of fine details in conventional feature transmission (Liu et al., 2018). However, this integrated structure has clear limitations. Its strong performance on specific datasets is difficult to transfer to other scenarios, and the model generalization ability declines markedly under complex environments, making it difficult to produce stable detection results (Zheng et al., 2024). In view of this, this study introduces BiFPN into the YOLOv9 architecture to replace the original combination of FPN and PANet. By leveraging the bidirectional cross-scale connections and dynamic weight allocation of BiFPN, the model enhances target localization accuracy and class discriminability in complex paddy-field scenarios, ultimately achieving improvements in both overall performance and cross-scenario adaptability.

**Figure 4:**
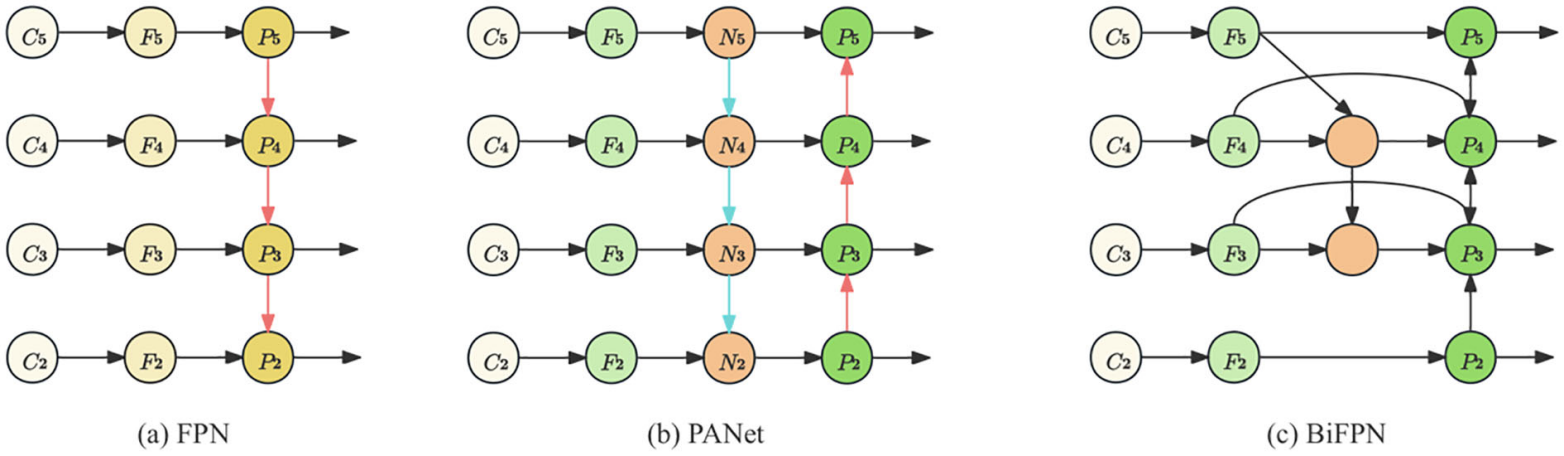
Structural comparison of feature fusion architectures: **(a)** Feature Pyramid Network (FPN); **(b)** Path Aggregation Network (PANet); and **(c)** Bidirectional Feature Pyramid Network (BiFPN).

From the perspective of structural characteristics, the information flow in FPN (**Figure 4a**) is unidirectional and only supports a top-down feature transmission pattern, making it difficult to fully exploit low-level feature information. To mitigate this limitation, PANet extends the original FPN architecture by introducing an additional bottom-up feature transmission path, thereby strengthening interlayer information interaction. Compared with the improvement strategy of PANet (**Figure 4b**), BiFPN further overcomes the limitations of path design by integrating bidirectional feature transmission paths into a repeatedly stackable feature network module, enabling deeper fusion of multi-dimensional features through module reuse. The structure of BiFPN (**Figure 4c**) consists of three key components: a top-down pathway responsible for semantic information transmission, a bottom-up pathway for positional information propagation, and weighted feature fusion with bidirectional cross-scale connections achieved through the coordination of skip connections and bidirectional pathways, thereby constructing a more efficient feature interaction framework.

During feature integration, BiFPN utilizes a weighted feature fusion strategy. Its main principle is to allocate a learnable weight to each input feature branch, while normalization is applied to avoid numerical instability caused by differences in branch magnitudes. The specific calculations are shown in (Equations 6, 7). To ensure that the fusion weights remain non-negative, a ReLU constraint is imposed on the learnable parameter ${\hat{\omega }}_{i}$ to obtain $\omega_{i}$. In addition, a small constant ∈ is introduced into the denominator to prevent numerical instability resulting from overly small denominator values. In (Equation 7), $I_{i}$ denotes the input feature map involved in feature fusion, and $\omega_{i}$ represents the learnable scalar weight corresponding to each branch.

(6) $$\omega_{i}=ReLU({\hat{\text{w}}}_{i})$$

(7) $$Fuse(I_{1},\ldots ,I_{n})=\frac{\sum_{i=1}^{n}\omega_{i}I_{i}}{\sum_{i=1}^{n}\omega_{i}+∊}$$

Using the sixth layer as an example, BiFPN generates intermediate features along the top-down pathway and output features along the bottom-up pathway, respectively, as expressed in (Equations 8, 9). Here, $P_{6}^{td}$ denotes the top-down intermediate feature transmitted from higher to lower layers, and $P_{6}^{out}$ denotes the bottom-up output feature transmitted from lower to higher layers. Resize represents the upsampling or downsampling operation, Conv denotes the convolution operation, and ϕ is the activation function introduced to provide nonlinearity to the features.

(8) $$P_{6}^{td}=\phi (Conv(Fuse(P_{6}^{in},Resize(P_{7}^{in}))))$$

(9) $$P_{6}^{out}=\phi (Conv(Fuse(P_{6}^{in},P_{6}^{td},Resize(P_{5}^{out}))))$$

### 2.6 RepFEL module

**Figure 5:**
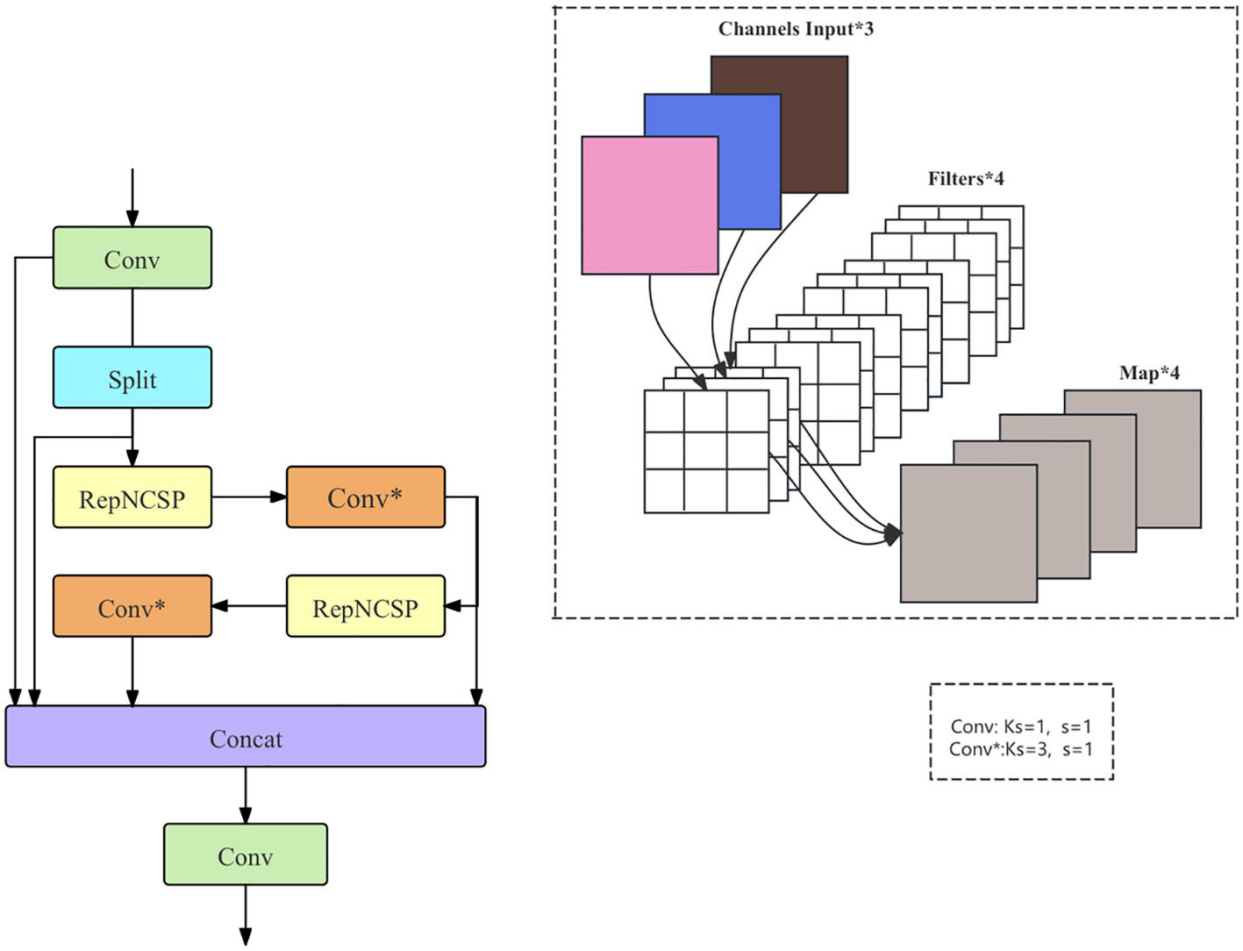
Architecture of the RepNCSPELAN4 module.

To strengthen the feature representation capability of the YOLOv9 model under complex paddy-field conditions, this study integrated a feature enhancement (FE) module with the large selective kernel attention mechanism (LSKBlock) and reconstructed the original RepNCSPELAN4 module (**Figure 5**), thereby developing the RepFEL module. RepNCSPELAN4 is a key component in the YOLOv9 architecture for feature extraction and hierarchical aggregation. Its design combines the advantages of CSPNet (Wang et al., 2020) and ELAN (Wang et al., 2022) to construct the GELAN, thus enhancing both detection accuracy and computational efficiency in object detection tasks. Within the RepNCSPELAN4 module, the convolutional layers (Conv) are primarily used for local feature extraction and channel transformation, while the RepNCSP branches further perform feature interaction and fusion to enhance feature representation and reduce redundant computation, thus forming an efficient feature processing pipeline (Balakrishnan and Sengar, 2024). On this basis, the RepFEL module introduces FE to strengthen input feature details and incorporates the large selective kernel mechanism of LSKBlock to enlarge the effective receptive field and enhance the response to key regions, thereby strengthening the model’s ability to detect targets in complex field environments.

**Figure 6:**
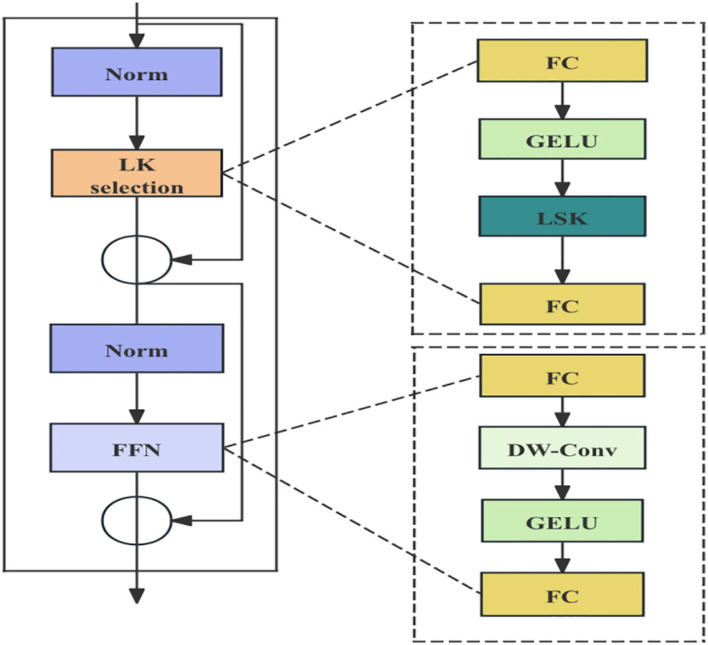
Structure of the LSKBlock module.

As shown in **Figure 6**, LSKBlock comprises two key submodules: the large kernel selection (LK selection) block and the feed-forward network (FFN) block (Li et al., 2023). The LK selection block allows the network to dynamically adapt its receptive field to target size and morphology, thereby improving its ability to handle features at different scales (Li et al., 2019). The FFN block enhances the nonlinear mapping capability of the network, helping the model learn more complex feature patterns (Qin et al., 2023). On this basis, an FE feature enhancement module was constructed in this study to enhance feature representation quality. The processing pipeline of the improved module can be divided into three stages. First, the input features undergo preliminary feature extraction and channel adjustment through convolutional layers (Woo et al., 2023). Second, the LSKBlock is introduced to enhance the response to key regions (Yu et al., 2024). Finally, convolutional layers are used again to remap the enhanced features to a channel dimension consistent with that of the backbone network, ensuring structural compatibility and effective feature integration with subsequent modules (Guo et al., 2023).

To quantitatively characterize changes in the receptive field of convolution operations within the LSKBlock branch, the concept of the effective kernel size was introduced into the dynamic large-kernel selection branch, and the receptive field of each layer was recursively calculated on this basis. $K_{i}$ denotes the kernel size of the i-th depthwise convolution, whereas $d_{i}$ denotes the corresponding dilation rate. Since dilated convolution enlarges the coverage range without substantially increasing the number of parameters, its equivalent receptive field is defined by the effective kernel size $k_{i}^{\text{eff}}$, as shown in Equation 10. To avoid parameter inflation caused by indefinitely increasing $k_{i}$, this study constrained the upper bound of $k_{i}$ and utilized $d_{i}$ to expand the receptive field, thereby achieving a balance between representation capability and computational complexity.

On this basis, let $RF_{i}$ denote the receptive field size corresponding to the output feature map of the i-th convolution operator. The receptive field is calculated recursively, as shown in (Equation 11). It should be noted that the convolutions in this branch generally use a stride of $s_{i}=1$; therefore, the recursive formulation can be written in a simplified form. For network layers involving downsampling, a jump stride term can be introduced into (Equation 10) to obtain a more general receptive field calculation. Based on the above quantitative mechanism, the model can adaptively allocate weights among multiple candidate branches through the selection weights of LSK, thereby enabling greater focus on local details for small targets and broader contextual coverage for large-scale targets. This improves feature representation and detection robustness under complex paddy-field backgrounds.

(10) $$k_{i}^{eff}=(k_{i}-1)d_{i}+1,$$

(11) $$RF_{0}=1,RF_{i}=RF_{i-1}+(k_{i}^{eff}-1)$$

**Figure 7:**
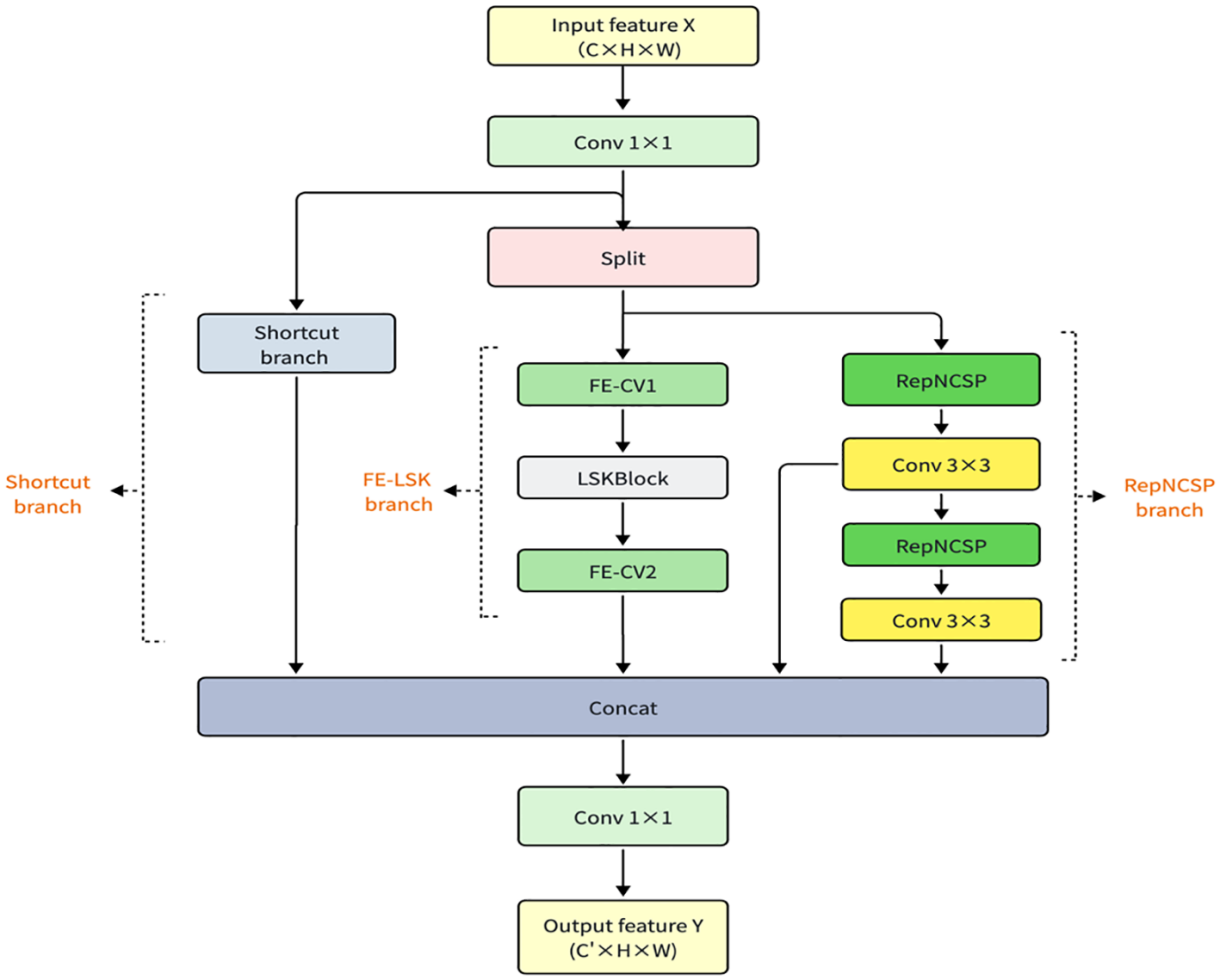
Structure of the RepFEL module.

**Figure 7** depicts the detailed structure of the RepFEL module. Given an input feature map $X\in {\text{R}}^{C\times H\times W}$, a 1×1 convolution is first applied to adjust the channel dimension and generate an intermediate feature U. A shortcut branch is directly connected from this transformed feature to the final concatenation layer to preserve the original feature representation. Meanwhile, the remaining feature flow is divided by the Split operation into two branches, namely the FE-LSK branch and the RepNCSP branch.

In the FE-LSK branch, the feature is first processed by FE-CV1 for local feature transformation and channel adjustment, and then fed into the LSKBlock. The LSKBlock adaptively adjusts the effective receptive field through its large-kernel selection mechanism, thereby enhancing the response to target-related regions while suppressing background noise. Subsequently, FE-CV2 remaps the enhanced feature for subsequent fusion. In the RepNCSP branch, two successive RepNCSP modules, each followed by a 3×3 convolution, are used to perform hierarchical feature interaction and aggregation. The intermediate output and final output of this branch are both connected to the concatenation layer.

Finally, the shortcut feature, the FE-LSK enhanced feature, and the two RepNCSP outputs are concatenated along the channel dimension and projected by a 1×1 convolution to obtain the output feature map $Y\in {\text{R}}^{C^{\prime }\times H\times W}$. Therefore, RepFEL is not a simple stacking of the FE module and LSKBlock, but a reconstruction of the original RepNCSPELAN4 feature aggregation path. By integrating original feature preservation, adaptive receptive-field enhancement, and RepNCSP-based hierarchical aggregation, RepFEL further reconstructs the multi-scale fused features output by BiFPN and improves detection stability in complex paddy-field scenarios.

### 2.7 BR-YOLOv9 module

**Figure 8:**
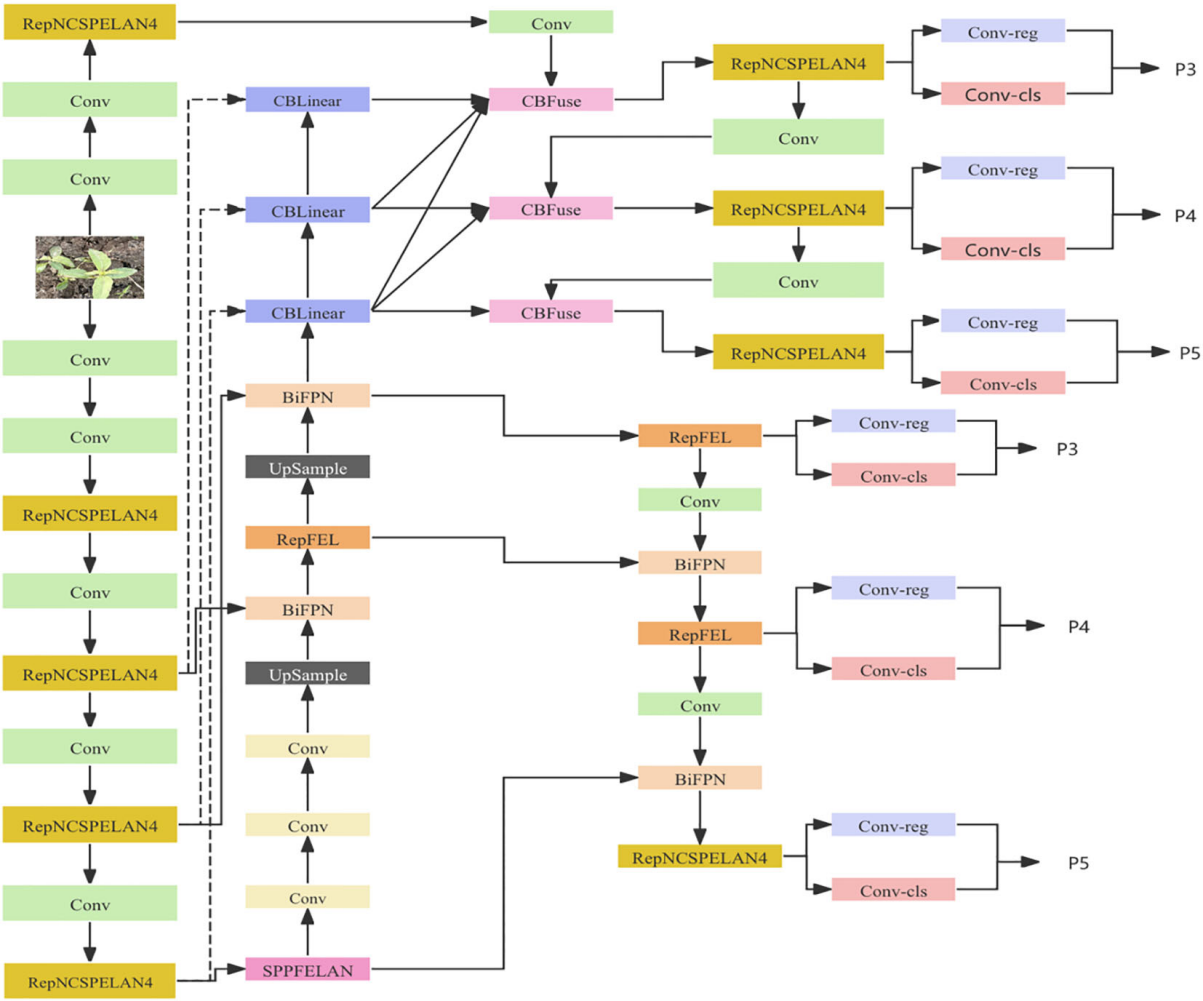
Architecture of the proposed BR-YOLOv9.

In this study, the RepFEL (Re-parameterized Feature-Enhanced LSK block) module was deeply integrated with the BiFPN module (Chen et al., 2021) to construct the BR-YOLOv9 object detection model (**Figure 8**). In the proposed BR-YOLOv9 architecture, RepFEL was not used to replace all RepNCSPELAN4 modules in YOLOv9. Instead, it was selectively introduced to replace the three RepNCSPELAN4 modules located in the neck network. This design was based on the functional difference between the backbone and neck structures. The RepNCSPELAN4 modules in the backbone mainly perform hierarchical feature extraction and maintain the original GELAN-based gradient flow and general feature representation capability of YOLOv9. Replacing all RepNCSPELAN4 modules would increase computational complexity and may affect the stability of the original backbone structure. In contrast, the RepNCSPELAN4 modules in the neck network are directly involved in multi-scale feature aggregation before the detection heads and have a more direct influence on the detection of small, medium, and large weed targets. Therefore, replacing only the three neck modules with RepFEL can enhance the multi-scale fused features generated by BiFPN, improve adaptive receptive-field modeling, and suppress background noise while maintaining the efficiency of the original backbone. The core rationale of this integrated design lies in the synergy between the two modules, which enables “multi-scale feature integration” and “key feature optimization,” respectively, thereby forming a progressive feature processing pipeline.

In the feature processing pipeline, BiFPN first serves as the multi-scale “feature aggregation core” of the network (Doherty et al., 2025). Through bidirectional feature transmission, this module simultaneously establishes top-down semantic information propagation and bottom-up detail information propagation, enabling full interaction between high-level semantics and low-level localization information. In addition, a learnable weighted fusion mechanism is incorporated to adaptively balance the contributions of features across various scales, rather than simply combining them with equal weights, thus enhancing the efficiency of multi-scale fusion while mitigating the loss of fine details resulting from unidirectional information flow (Shao et al., 2025). The multi-level “semantic–detail” features aggregated by BiFPN provide a more sufficient and robust input representation for the subsequent RepFEL module.

Following the BiFPN module, the RepFEL module focuses on improving feature quality. On the one hand, it applies a feature enhancement strategy to strengthen pixel-level details in the input multi-scale features, thereby highlighting discriminative differences between targets and the background (Wang et al., 2024). On the other hand, it introduces the LSKBlock attention mechanism, which exploits its capacity to model long-range dependencies, thereby adaptively attending to key target regions, such as the edge features of small-scale weeds, while suppressing noise caused by complex backgrounds, including illumination variation in the field and overlapping regions between weeds and rice plants (Lau et al., 2024).

Through the synergy of the above modules, the BR-YOLOv9 model achieves a closed-loop process of “feature integration–feature optimization.” The BiFPN module provides the model with a rich multi-scale feature foundation, while the RepFEL module further enhances feature discriminability on this basis. As a result, the model simultaneously improves its robustness to complex field environments and its ability to accurately identify small-scale targets, thereby providing a technical foundation for subsequent target detection tasks in precision agriculture.

## 3 Results and analysis

### 3.1 Ablation experiments

To assess the contributions of the BiFPN and RepFEL modules to the weed detection model, this study selected YOLOv9 as the baseline network architecture and designed ablation experiments accordingly (Li et al., 2025). In addition to detection accuracy, model computational complexity was also considered to evaluate the practicality of the proposed improvements. Specifically, mAP@0.5 and mAP@0.5:0.95 were used to evaluate detection performance, while Params and GFLOPs were introduced to quantify model size and computational cost, respectively. Params reflects the number of learnable parameters and indicates the storage requirement of the model, whereas GFLOPs measures the computational complexity of a single forward pass under the same input resolution. The corresponding ablation results and complexity comparisons are shown in **Table 2**.

**Table 2:** Results of the ablation experiments.

YOLOv9	BiFPN	FE	LSK	mAP@0.5	mAP@0.5:0.95	GFLOPs	Params/M	FPS
✓	—	—	—	96.18	92.26	19.80	7.14	324.13
✓	✓	—	—	96.42	92.72	21.16	7.65	313.75
✓	—	✓	—	96.35	92.61	20.36	7.31	315.28
✓	—	—	✓	96.37	92.62	20.53	7.42	312.46
✓	—	✓	✓	97.08	93.84	22.18	7.95	310.59
✓	✓	✓	✓	98.21	95.97	23.41	8.43	304.97

“✓” denotes the inclusion of the corresponding component, whereas “—” denotes its absence.

As shown in **Table 2**, after integrating the BiFPN weighted bidirectional feature pyramid network into the model, mAP@0.5 and mAP@0.5:0.95 increased by 0.24 and 0.46 percentage points, respectively, compared with the baseline YOLOv9 model. This indicates that weighted bidirectional multi-scale feature fusion can enhance the representation of weed targets under complex paddy-field conditions. When the FE and LSK modules were introduced, mAP@0.5 and mAP@0.5:0.95 increased by 0.90 and 1.58 percentage points, respectively, suggesting that feature enhancement and adaptive large-kernel receptive-field selection improved the model’s ability to detect weeds with scale variation and background interference. Among all configurations, the complete BR-YOLOv9 model achieved the best detection accuracy, with mAP@0.5 and mAP@0.5:0.95 reaching 98.21% and 95.97%, respectively, corresponding to improvements of 2.03 and 3.71 percentage points over the original YOLOv9 model.

**Figure 9:**
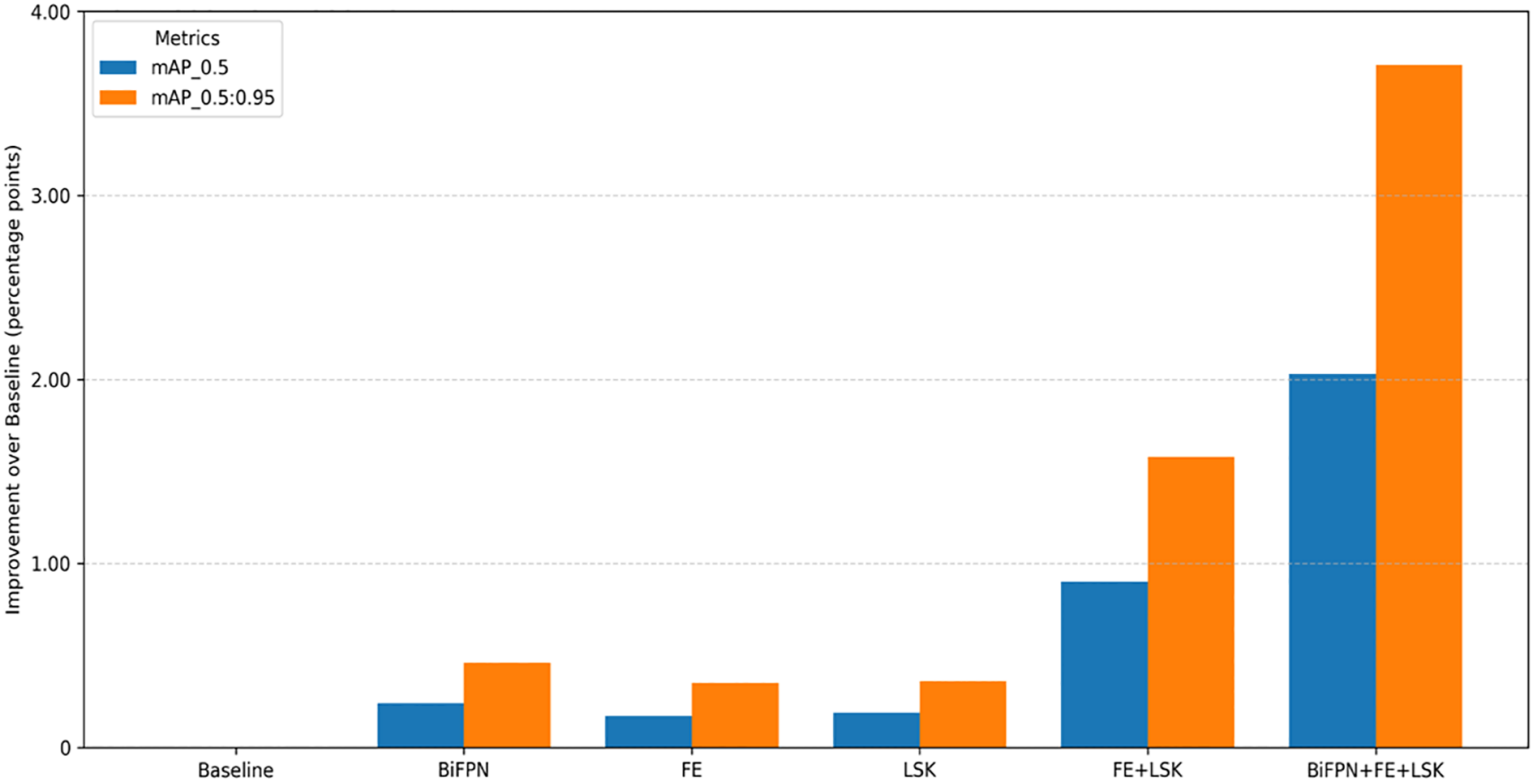
Bar chart of ablation study results.

In addition to detection accuracy, Params, GFLOPs, and FPS were introduced in **Table 2** to evaluate the model size, computational cost, and inference efficiency of different module combinations. The results show that the introduction of BiFPN, FE, and LSKBlock increased model complexity to varying degrees. Compared with the baseline YOLOv9 model, the complete BR-YOLOv9 increased GFLOPs from 19.80 G to 23.41 G and Params from 7.14 M to 8.43 M, while FPS decreased from 324.13 to 304.97. Although the computational cost increased, BR-YOLOv9 still maintained a high inference speed and achieved the highest detection accuracy among all configurations. Therefore, the ablation results demonstrate that the proposed modules provide an effective accuracy, complexity and speed trade-off. **Figure 9** visualizes the improvements in mAP@0.5 and mAP@0.5:0.95 over the baseline model under different component combinations, further confirming that the combined use of BiFPN, FE, and LSKBlock contributes most significantly to performance improvement.

### 3.2 Comparative experiments

**Figure 10:**
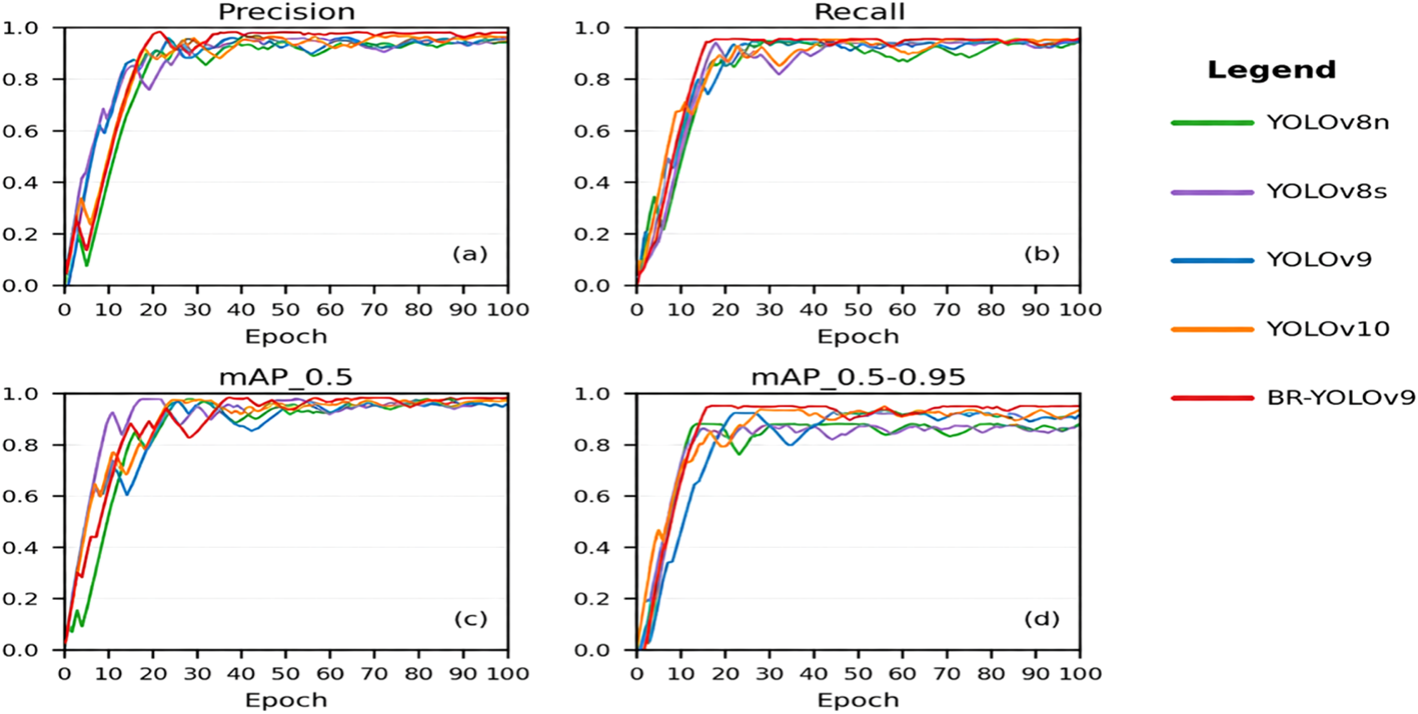
Comparison of model performance on the test set.

To further examine the weed recognition performance, model complexity, and inference efficiency of the proposed method, BR-YOLOv9 was trained and tested alongside YOLOv8n, YOLOv8s, YOLOv9, and YOLOv10 under the same experimental conditions and using the same dataset. In addition to precision, recall, mAP@0.5, and mAP@0.5:0.95, GFLOPs, Params, and FPS were introduced to evaluate the computational cost, model size, and inference speed of different detectors. **Table 3** summarizes the comparative results. As shown in **Table 3** and **Figure 10**, BR-YOLOv9 achieved the highest detection accuracy among the compared models, with precision, recall, mAP@0.5, and mAP@0.5:0.95 reaching 98.04%, 95.65%, 98.21%, and 95.97%, respectively.

**Table 3:** Comparative results of detection performance and model complexity.

Models	Precision	Recall	mAP@0.5	mAP@0.5:0.95	GFLOPs	Params/M	FPS
YOLOv8s	95.71	94.36	97.65	87.43	21.45	11.47	314.91
YOLOv8n	94.27	94.72	97.33	87.36	6.53	3.22	398.21
YOLOv9	96.23	94.46	96.18	92.26	19.80	7.14	324.13
YOLOv10	96.62	95.41	97.12	93.45	16.24	7.26	343.98
BR-YOLOv9	98.04	95.65	98.21	95.97	23.41	8.43	304.97

Compared with YOLOv9, BR-YOLOv9 improved mAP@0.5 and mAP@0.5:0.95 by 2.03 and 3.71 percentage points, respectively. Meanwhile, GFLOPs increased from 19.80 G to 23.41 G, Params increased from 7.14 M to 8.43 M, and FPS decreased from 324.13 to 304.97. Although the proposed model introduces additional computational cost due to the integration of BiFPN and RepFEL, it still maintains a high inference speed while achieving the best detection accuracy. These results indicate that BR-YOLOv9 improves detection accuracy and robustness under complex paddy-field conditions with a moderate increase in computational complexity, showing a favorable accuracy–complexity–speed trade-off.

### 3.3 Per-class performance and confusion matrix analysis

As shown in **Table 4**, BR-YOLOv9 achieved high AP values for all weed categories, with mean AP@0.5 and AP@0.5:0.95 values of 98.21% and 95.97%, respectively. Among them, categories such as Portulaca oleracea, Monochoria vaginalis, and Cirsium setosum obtained relatively high AP values, indicating that the model can effectively recognize weed species with distinctive morphological characteristics. In contrast, some narrow-leaf weed species, such as Digitaria sanguinalis, Leptochloa chinensis, Setaria viridis, and Echinochloa crus-galli, showed slightly lower AP values. This may be attributed to their similar leaf morphology, color, texture, and growth posture, which make fine-grained discrimination more challenging under complex paddy-field conditions.

**Table 4:** Per-class results of BR-YOLOv9.

Weed species	AP@0.5	AP@0.5:0.95
Eleusine indica	98.64	96.41
Eclipta prostrata	98.47	96.33
Alternanthera sessilis	98.21	95.94
Portulaca oleracea	98.70	96.37
Echinochloa crus-galli	97.79	95.29
Setaria viridis	97.92	95.45
Digitaria sanguinalis	97.76	95.71
Leptochloa chinensis	97.83	95.82
Monochoria vaginalis	98.58	96.68
Erigeron canadensis	98.31	96.15
Kalimeris indica	98.17	96.23
Cirsium setosum	98.14	96.62
Mean	98.21	95.97

**Figure 11:**
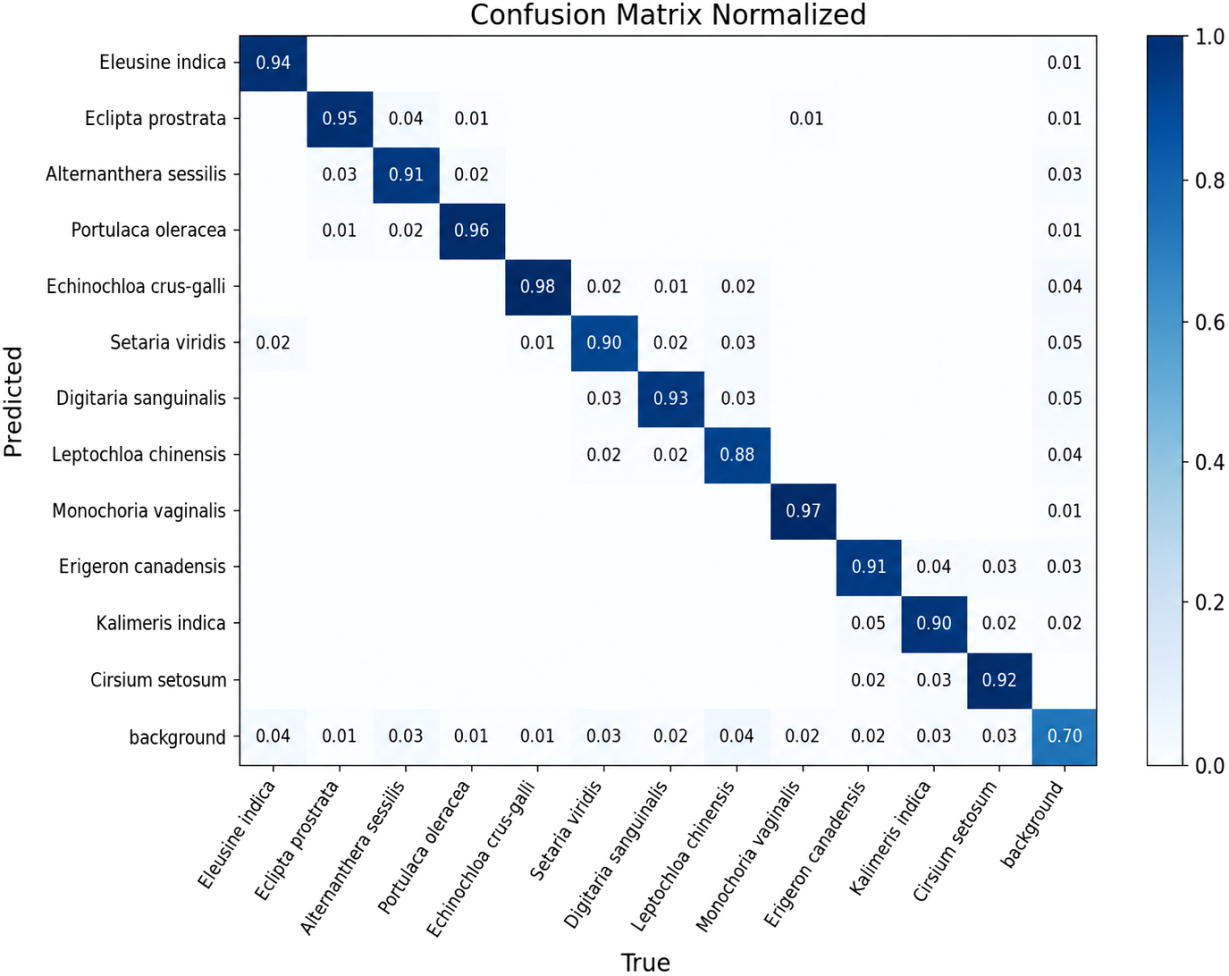
Confusion matrix of BR-YOLOv9.

The normalized confusion matrix in **Figure 11** shows that most samples were concentrated along the diagonal, indicating that BR-YOLOv9 has strong overall category discrimination capability. However, several off-diagonal elements were still observed among visually similar categories. For example, slight confusion occurred among narrow-leaf weeds such as Setaria viridis, Digitaria sanguinalis, Leptochloa chinensis, and Echinochloa crus-galli. In addition, minor confusion was observed between some broadleaf weed categories, such as Eclipta prostrata, Alternanthera sessilis, and Portulaca oleracea, as well as between Erigeron canadensis and Kalimeris indica. These misclassifications may be related to similar leaf shapes, overlapping growth, partial occlusion, and uneven illumination in field images.

The background-related entries in the confusion matrix indicate that a small number of weed targets were confused with background regions, or that background regions were incorrectly predicted as weed categories. This phenomenon is mainly caused by complex paddy-field interference, such as soil spots, water reflections, dense vegetation, and fragmented leaf textures. Overall, the per-class AP values and confusion matrix demonstrate that BR-YOLOv9 achieves strong multi-class weed recognition performance, while fine-grained discrimination among morphologically similar weed species remains a challenging issue.

### 3.4 Comparison of visual detection results

**Figure 12:**
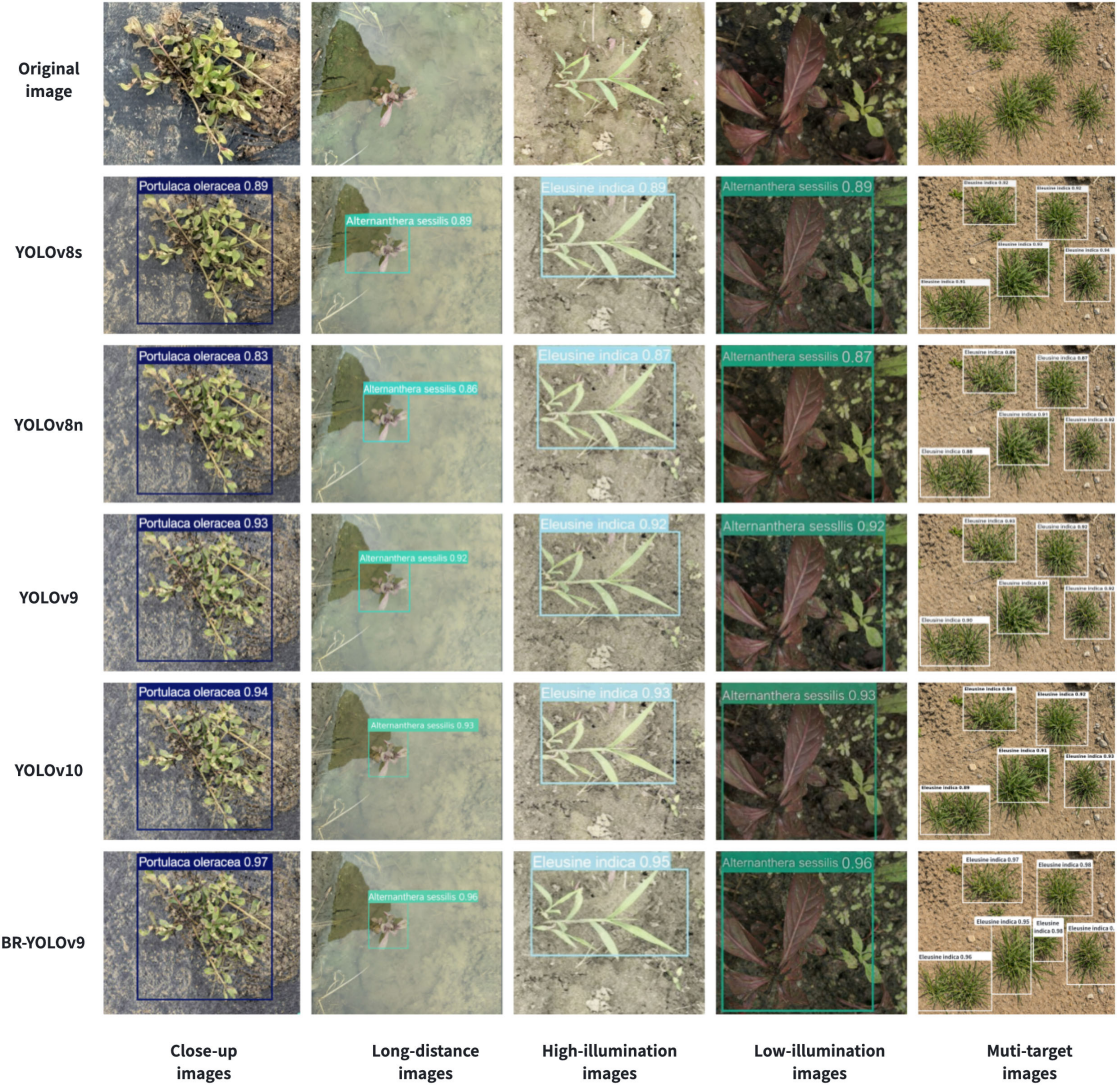
Qualitative comparison of detection performance among different models.

As shown in **Figure 12**, under task scenarios such as close-up, long-distance, high-illumination, low-illumination, and multi-target conditions, BR-YOLOv9 exhibited higher detection accuracy and more stable performance than mainstream models, including YOLOv8s, YOLOv8n, YOLOv9, and YOLOv10. These results indicate that BR-YOLOv9 can not only localize targets more precisely, but also produce more reasonable and reliable confidence distributions in complex and dynamic field environments, thereby improving the robustness of detection.

**Figure 13:**
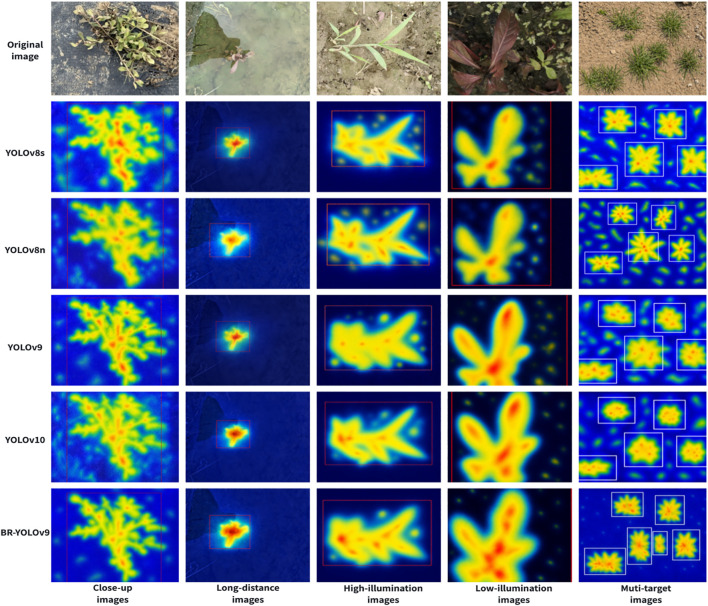
Heatmap visualization comparison of different models for paddy-field weed detection.

To further intuitively compare the feature perception ability of different models, heatmap visualization was introduced to analyze the attention regions during weed detection. As shown in **Figure 13**, the first row presents the original images under different paddy-field conditions, and the following rows show the corresponding heatmap responses of different models. The visualization results show that the compared models can generally focus on weed target regions, but some models still produce scattered activation responses in background areas, especially under complex conditions such as water reflection, soil texture interference, dense weed distribution, and small target scenes. In contrast, BR-YOLOv9 exhibits more concentrated and complete activation responses on weed targets. The high-response regions are more consistent with the actual weed contours and bounding boxes, indicating that the proposed model can better capture discriminative weed features while reducing attention to irrelevant background regions. This improvement can be attributed to the combined effect of BiFPN and RepFEL. BiFPN strengthens multi-scale feature fusion and helps preserve spatial information of small and multi-scale weed targets. RepFEL further enhances local feature representation and adaptive receptive-field selection through FE and LSKBlock, allowing the model to focus more effectively on target-related regions. Therefore, the heatmap visualization further verifies that BR-YOLOv9 improves feature extraction capability and robustness under complex paddy-field backgrounds.

### 3.5 Generalization and external validation experiments

#### 3.5.1 Scenario-based generalization evaluation

**Figure 14:**
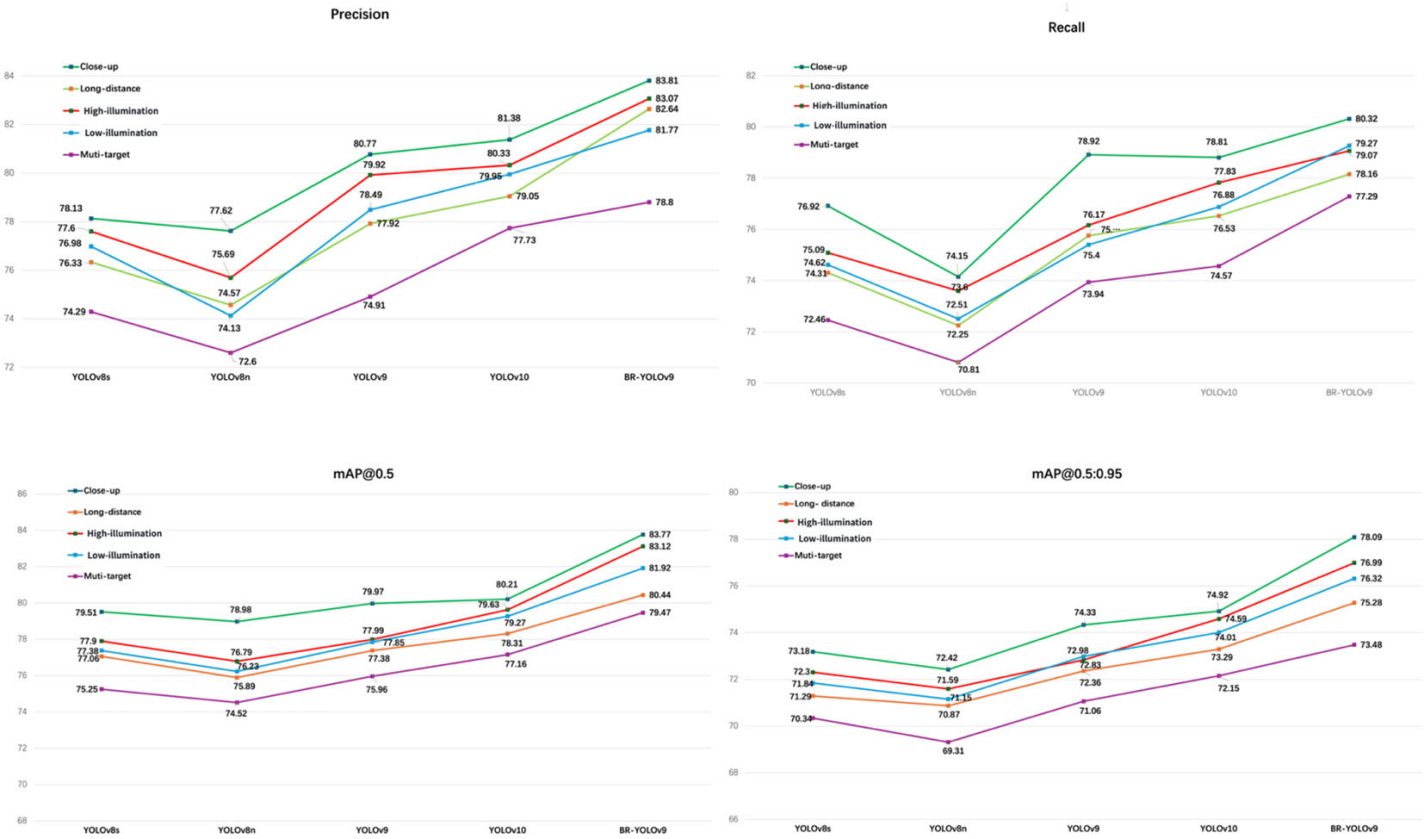
Generalization performance of different models under different field scenarios.

To assess the generalization performance of the BR-YOLOv9 model, the iNaturalist dataset (López-Guillén et al., 2024) was used for testing in this section. This dataset contains high-quality images of 12 common paddy-field weeds, including Eleusine indica, Eclipta prostrata, Alternanthera sessilis, and Portulaca oleracea. A total of 2,000 images were randomly selected for testing. In order to more intuitively evaluate the robustness of different models under diverse field conditions, the test images were divided into five representative scenario subsets, namely close-up, long-distance, high-illumination, low-illumination, and multi-target scenes. The corresponding precision, recall, mAP@0.5, and mAP@0.5:0.95 values are illustrated in **Figure 14**. The figure shows the performance variation of each model across different scenario subsets and provides a more direct comparison of their generalization ability under changes in imaging distance, illumination, and scene complexity.

As shown in **Figure 14**, BR-YOLOv9 achieved the best overall performance across the five scenario subsets, including close-up, long-distance, high-illumination, low-illumination and multi-target scenes. The results indicate that BR-YOLOv9 maintains stronger robustness than the compared models under variations in imaging distance, illumination, and target density.

The performance of all models was relatively higher under close-up and high-illumination conditions, where weed targets had clearer contours and richer visual details. By contrast, low-illumination and multi-target scenes caused more obvious performance degradation because of reduced image contrast, overlapping leaves, and increased background complexity. Nevertheless, BR-YOLOv9 maintained higher precision, recall, mAP@0.5, and mAP@0.5:0.95 than the other models, demonstrating stronger adaptability to complex paddy-field environments. This improvement is mainly attributed to the enhanced multi-scale feature fusion of BiFPN and the feature reconstruction capability of RepFEL.

#### 3.5.2 Complex-background qualitative validation on the self-constructed test set

**Figure 15:**
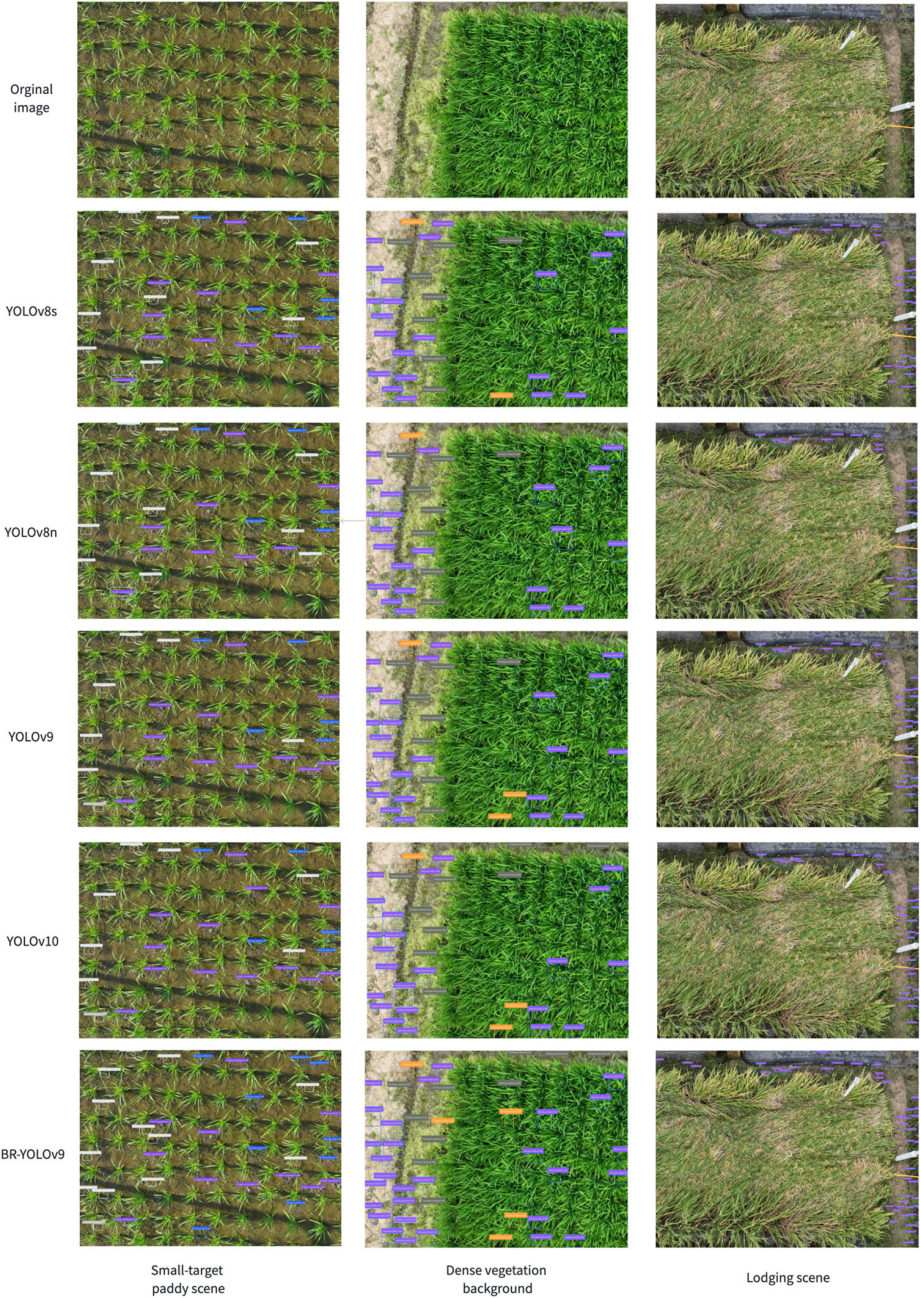
Qualitative comparison of detection results under complex top-view paddy-field backgrounds.

To further demonstrate the superiority of BR-YOLOv9 under more complex paddy-field environments, representative complex-background samples were selected from the independent test set for qualitative comparison. As shown in **Figure 15**, these top-view paddy-field images include muddy-water backgrounds, dense vegetation, rice–weed visual similarity, partial occlusion, and lodging interference. Compared with YOLOv8s, YOLOv8n, YOLOv9, and YOLOv10, BR-YOLOv9 produced more stable and complete detection results under these challenging conditions, with fewer missed detections and more accurate localization. These results further indicate that the proposed model has superior robustness and adaptability in complex paddy-field weed detection scenarios.

#### 3.5.3 External validation on a public weed dataset

To further evaluate the cross-domain generalization ability of the proposed model, an external validation experiment was conducted on the CropAndWeed public dataset. As shown in **Table 5**, all models showed lower detection performance on the external dataset than on the self-constructed paddy-field test set, which can be attributed to domain shifts in crop type, weed species, plant morphology, field background, illumination conditions, and annotation distribution. Nevertheless, BR-YOLOv9 achieved the best overall performance among all compared models, with Precision, Recall, mAP@0.5, and mAP@0.5:0.95 values of 80.17%, 75.86%, 74.92%, and 65.24%, respectively. Compared with the baseline YOLOv9, BR-YOLOv9 improved Precision, Recall, mAP@0.5, and mAP@0.5:0.95 by 4.65, 2.99, 5.81, and 7.31 percentage points, respectively. These results indicate that the proposed BiFPN-based multi-scale feature fusion and RepFEL-based feature enhancement modules improve the robustness and cross-domain generalization ability of weed detection under complex agricultural field conditions. The external validation results further demonstrate that BR-YOLOv9 does not merely fit the self-constructed paddy-field dataset, but also maintains stronger robustness on a public crop–weed dataset with more diverse field backgrounds.

**Table 5:** External validation results on the CropAndWeed public dataset.

Models	Precision	Recall	mAP@0.5	mAP@0.5:0.95	GFLOPs	Params/M	FPS
YOLOv8s	74.73	70.21	68.59	54.27	21.45	11.47	302.78
YOLOv8n	73.68	70.13	65.96	52.15	6.53	3.22	374.22
YOLOv9	75.52	72.87	69.11	57.93	19.80	7.14	307.65
YOLOv10	76.39	73.35	69.30	59.79	16.24	7.26	326.86
BR-YOLOv9	80.17	75.86	74.92	65.24	23.41	8.43	297.04

### 3.6 In-field weed recognition system

**Figure 16:**
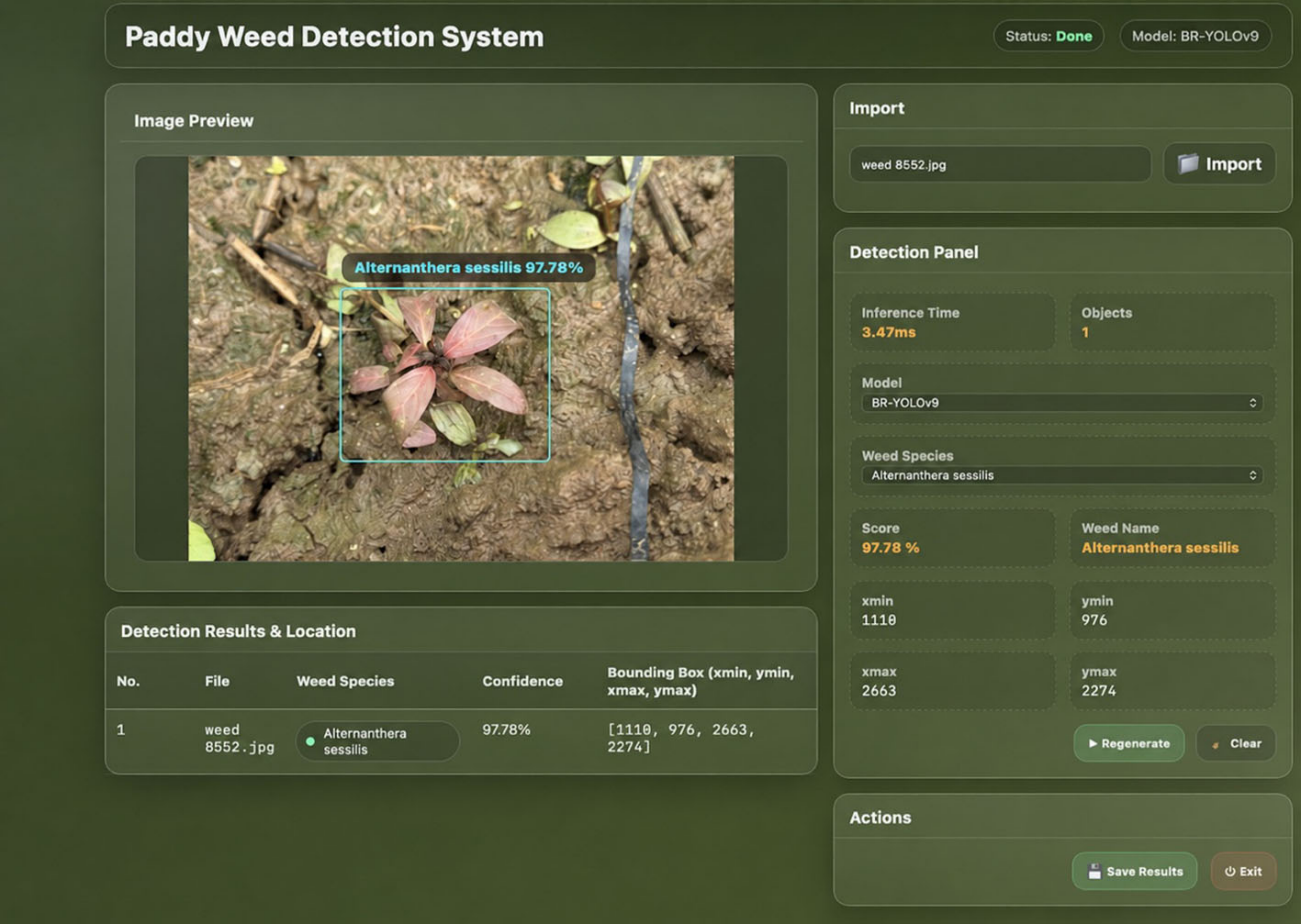
Main interface of the developed rice-paddy weed recognition system.

**Figure 17:**
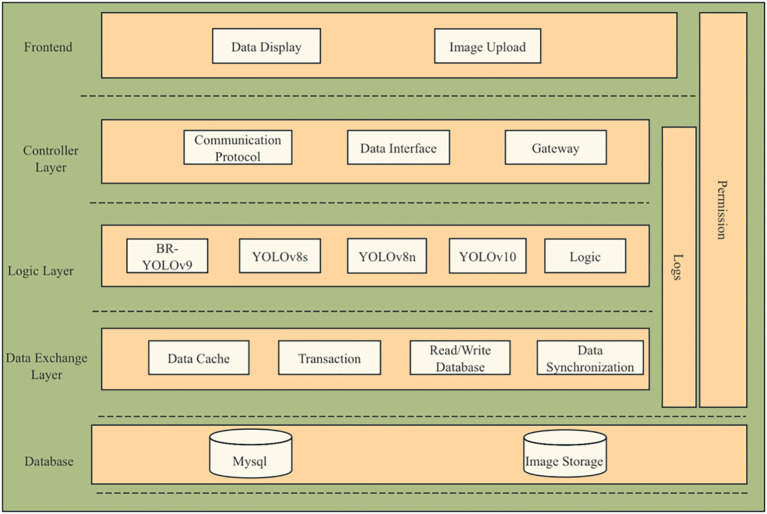
System architecture of the proposed weed recognition system.

To enable more intuitive identification of the recognized weed species, this study designed a weed recognition system (**Figure 16**). The system was developed based on MySQL8.0, Python 3.11, and PyQt5, and its architecture follows the classic MVC (Model–View–Controller) three-layer design pattern (**Figure 17**). The presentation layer supports user upload of weed images and displays the recognition results. The control layer is responsible for communication between the presentation layer and the business logic layer, including communication protocols, data interfaces, and gateways. The business logic layer is responsible for the implementation of system functions and the construction of the weed recognition algorithm model. Finally, the data exchange layer is responsible for database read and write operations.

To further evaluate the usability of the developed weed recognition system, preliminary system-level tests were conducted in terms of overall response speed, recognition module efficiency, operational stability, functional completeness, and API availability. Under the same GPU-based experimental environment, the BR-YOLOv9 recognition module achieved an average end-to-end recognition time of 3.47 ms per image, corresponding to a high inference efficiency for weed detection. Repeated recognition tests showed that the system could complete image input, model invocation, detection result visualization, and user interaction normally without abnormal interruption during the testing process. In terms of functional verification, the prototype successfully supported image upload, weed detection, result display, and basic user interaction. In addition, Postman was used to test the backend API interface, including image transmission, request response, and recognition result return. The API test results showed that the backend service could correctly receive input images and return detection results in the expected format. These results indicate that the developed software prototype can support the complete workflow of weed image input, intelligent recognition, and result visualization. However, the current system test was conducted on a GPU-based experimental platform, and the prototype has not yet been fully integrated with embedded edge devices or real weeding actuators. Therefore, further field deployment tests are still required to evaluate long-term operational stability and device-level integration performance under real paddy-field conditions.

## 4 Discussion

Weed recognition in paddy fields remains challenging because of complex field conditions, including illumination variation, long-distance imaging, crop–weed occlusion, multi-scale targets, and background interference. Although traditional machine-learning methods based on handcrafted features have been used for crop–weed discrimination, their robustness is limited under natural field environments (Ferreira et al., 2017). Deep-learning-based detectors have substantially improved weed recognition performance; however, unstable detection accuracy and limited generalization ability still occur when weed targets are small, partially occluded, or distributed in complex backgrounds. To address these challenges, this study proposed BR-YOLOv9 by integrating BiFPN and RepFEL into the YOLOv9 framework. The purpose was to enhance multi-scale feature fusion, improve fine-grained weed feature representation, and suppress irrelevant background noise, thereby improving the robustness of paddy-field weed detection under complex natural conditions.

The main contribution of this study is the development of a YOLOv9-based paddy-field weed detection framework that directly addresses the key challenges raised in the Introduction, including multi-scale weed targets, illumination variation, vegetation occlusion, and complex background interference. By integrating BiFPN and RepFEL into YOLOv9, the proposed BR-YOLOv9 model strengthens cross-scale feature fusion, enhances fine-grained weed feature representation, and suppresses irrelevant background responses, thereby improving the robustness of weed recognition under natural paddy-field conditions.

Compared with previous weed detection studies that mainly relied on feature pyramid structures, attention mechanisms, or conventional YOLO-based improvements, BR-YOLOv9 combines weighted bidirectional multi-scale aggregation with adaptive receptive-field enhancement (Fan et al., 2024). This progressive feature optimization strategy enables the model to better capture both local weed details and broader contextual information, which is particularly important for detecting small, overlapping, or partially occluded weeds in complex field environments (Guo et al., 2024). Therefore, the proposed method provides a more task-oriented visual perception solution for intelligent weeding and precision herbicide application.

Nevertheless, this study still has several limitations. First, the experimental data mainly focused on rice paddy-field scenarios, and the adaptability of BR-YOLOv9 to other crop systems, such as wheat, soybean, and maize fields, requires further validation. Second, although the model maintained high inference efficiency, the introduction of BiFPN and RepFEL increased model complexity to some extent, which may affect deployment on low-cost embedded devices. Third, the current comparison mainly focused on representative YOLO-series models, while broader comparisons with classic CNN-based detectors, Transformer-based detectors, and newer YOLO variants remain to be conducted under a unified training and evaluation framework.

Overall, the most important contribution of this study is that BR-YOLOv9 improves weed detection accuracy and robustness by jointly enhancing multi-scale feature fusion and anti-interference feature representation. This confirms that the proposed YOLOv9-based improvement strategy is effective for complex paddy-field weed recognition, although further validation is still needed for broader agricultural scenarios.

Future research will focus on three specific directions. First, more field images from different crops, regions, growth stages, and environmental conditions will be collected to evaluate the cross-crop and cross-scenario generalization ability of BR-YOLOv9. Second, lightweight compression strategies, such as pruning, knowledge distillation, and lightweight convolutional modules, will be explored to reduce computational cost and facilitate deployment on edge devices. Third, a more comprehensive benchmark will be established by comparing BR-YOLOv9 with other advanced detectors under consistent experimental settings.

## 5 Conclusion

This study proposed an improved YOLOv9-based model, BR-YOLOv9, for paddy-field weed detection under complex natural conditions involving multi-scale targets, illumination variation, crop–weed occlusion, and background interference. By integrating BiFPN and the proposed RepFEL module, the model enhanced multi-scale feature fusion, fine-grained weed feature representation, and background-noise suppression. Experimental results showed that BR-YOLOv9 achieved mAP@0.5 and mAP@0.5:0.95 values of 98.21% and 95.97%, respectively, which were 2.03 and 3.71 percentage points higher than those of the original YOLOv9 model. These results demonstrate that the proposed improvements effectively enhance the detection accuracy and robustness of YOLOv9 in complex paddy-field environments. In the generalization experiments, BR-YOLOv9 was further evaluated under multiple representative field scenarios, including close-up, long-distance, high-illumination, low-illumination, and multi-target conditions. The results indicated that the proposed model maintained stable detection performance across different imaging distances, illumination conditions, and target densities, demonstrating stronger robustness and generalization capability in complex agricultural environments. In addition, BR-YOLOv9 maintained high inference efficiency while improving detection accuracy, indicating a favorable balance between recognition performance and real-time application potential. Overall, the proposed BR-YOLOv9 model provides an accurate and robust visual perception method for paddy-field weed detection. It can provide technical support for precise weed localization, precision herbicide application, and intelligent weeding equipment. However, further lightweight optimization and field deployment tests on embedded platforms are still needed to reduce computational cost and improve practical applicability in real agricultural production environments.

## Data Availability

The raw data supporting the conclusions of this article will be made available by the authors, without undue reservation.
